# Terebinthinated Caoutchouc in Phthisis

**Published:** 1861-07

**Authors:** 


——Pure Caoutchouc, dissolved in essential oil of turpentine and made into an electuary with syrup of elder and a few drops of essential oil of bitter almonds, is highly recommended by Prof. Hannon in phthisis in doses of four teaspoonsful of the electuary per diem, each teaspoonful containing about fifteen grains of the terebinthinated caoutchouc. Two spoonsful are given in the forenoon, and two in the afternoon, with an interval of two hours between each. "The dose may be gradually increased, as the stomach can bear, and the patient gets accustomed to the taste and smell of the turpentine to a drachm, or even a drachm and a half, of the terebinthinated caoutchouc. This administration must be continued daily, till the symptoms of pulmonary phthisis disappear; and I do not abandon it even then entirely, but simply decrease the dose."

Caoutchouc administered in the solid state is inert; it is not digested; it traverses the intestinal canal without alteration; but after "disaggregation," by means of the turpentine, it is easily digested, and seems to favor hematosis considerably. Chemically, no *respiratory food* can be richer in carbon and hydrogen than pure caoutchouc, which consists of only these elements.

In phthisical patients, "under the influence of terebinthinated caoutchouc, expectoration diminishes rapidly; oppression ceases; night-sweats disappear; diarrhœa and fever stop; gradually strength reappears, and emaciation gives way to *embonpoint.* The cough is one of the first symptoms, too, which disappears.

"No other appropriate treatment need be excluded, while giving the caoutchouc, but it is indispensable to give the latter through the whole continuance of treatment. That the cure of phthisis is not beyond the power of nature, very numerous examples prove; and that the terebinthinated caoutchouc, combined with other remedies, will assist in bringing about such a result, many observations lead me to believe."

Dr. HANNON refers to about a dozen cases.

